# Supplementary material for: Efficacy and Safety of Oral Administration of Wine Lees Extract (WLE)-Derived Ceramides and Glucosylceramides in Enhancing Skin Barrier Function: A Randomized, Double-Blind, Placebo-Controlled Study
Source: Nutrients. 2024 Jul 1;16(13):2100. doi: 10.3390/nu16132100 (PMC11243426; doi:10.3390/nu16132100)
Supplement: Supplementary file 1 [file nutrients-16-02100-s001.zip › nutrients-3082661-supplementary.pdf]

Figure S1. TLC of isolated ceramides and glucosylceramides from wine lees compared with standard *Citrus unshiu* ceramides and konjac glucosylceramides.

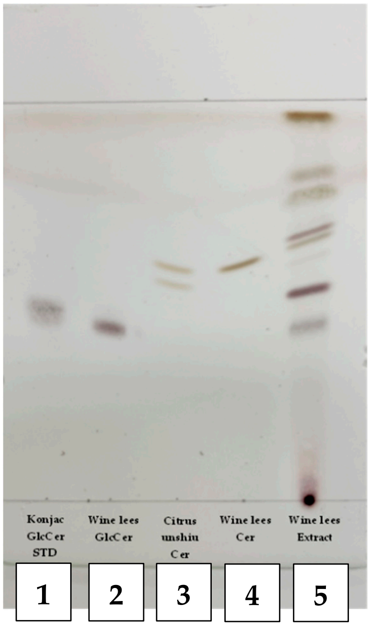

Thin Layer Chromatography (TLC), Plate: Silica gel 60, Eluent: Chloroform:Methanol:Water (65 : 15 : 2), Visualized using 5% H_2SO_4 in Ethanol. (1) konjac glucosylceramides STD, (2) wine lees glucosylceramides, (3) *Citrus unshiu* ceramides STD, (4) wine lees ceramides (5) wine lees extract.

Figure S2. HPLC chromatogram of isolated ceramides, glucosylceramides and WLE.

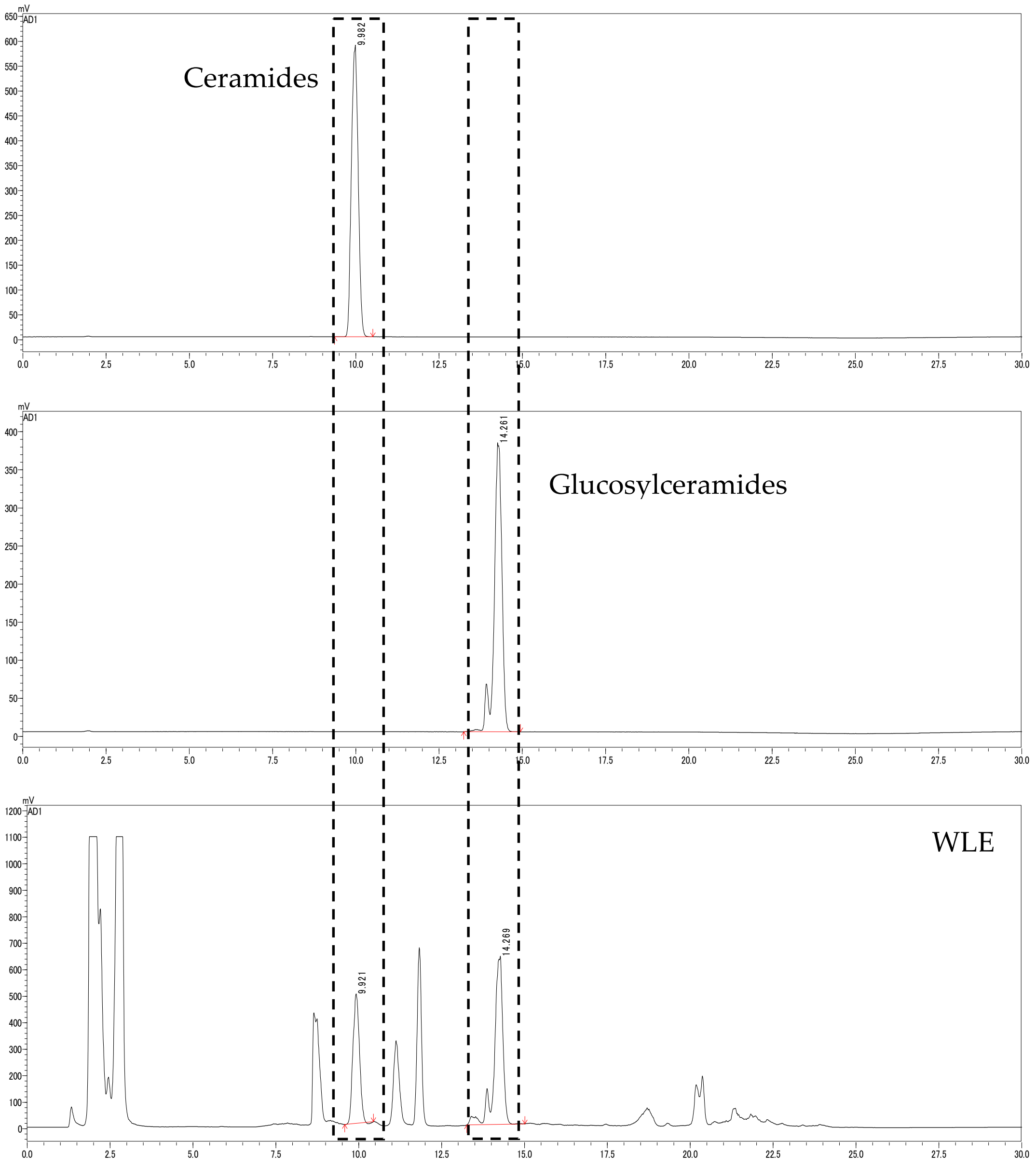

Table S1. List of primers and the primer sequences

Oligo Name	Sequences
Cers1-F	ACGCTACGCTATACATGGACAC
Cers1-R	AGGAGGAGACGATGAGGATGAG
Cers3-F	ACATTCCACAAGGCAACCATTG
Cers3-R	CTCTTGATTCCGCCGACTCC
SPTLC 2-F	CCAGACTGTCAGGAGCAACCATTA
SPTLC2-R	CGTGTCCGAGGCTGACCATA
PFR-F	GGCACTGAAAGGCAAAAAG
PFR-R	AAACCCGGATTCACCATAATC
INV-F	CTGCCTCAGCCTTACTGTGA
INV-R	GGAGGAACAGTCTTGAGGAG
LOR-F	GCAAACCTCGGGTAGCATCA
LOR-R	GCCGTCCAAATAGATCCCCC
GAPDH-F	GCACCGTCAAGGCTGAGAAC
GAPDH-R	TGGTGAAGACGCCAGTGGA

Table S2. Biochemical parameters from blood samples of participants

	Normal Range	Group	0w	4w	8w	12w	16w
			Mean ± SD	Mean ± SD	Mean ± SD	Mean ± SD	Mean ± SD
AST (U/L)	10-40	Test	23.7 ± 8.1	22.5 ± 6.7	20.1 ± 7.4	22.8 ± 9.3	21.7 ± 7.4
		Placebo	24.6 ± 12.4	21.7 ± 8.6	23.4 ± 11.4	19.6 ± 4.7	19.8 ± 5.2
ALT (U/L)	5-40	Test	26.4 ± 15.8	27.1 ± 15.8	22.1 ± 13.9	31.9 ± 42.1	22.1 ± 11.3
		Placebo	26.0 ± 19.0	21.2 ± 11.3	20.3 ± 9.0	18.6 ± 7.7	18.6 ± 9.0
γ-GT	F: -30	Test	43.6 ± 41.7	43.1 ± 37.2	42.2 ± 40.8	46.8 ± 48.8	39.4 ± 29.1
(U/L)	M: -70	Placebo	26.1 ± 16.1	22.7 ± 7.0	25.4 ± 12.9	25.3 ± 11.9	26.1 ± 17.9
LDH (U/L)	124-222	Test	168.5 ± 25.7	166.9 ± 22.0	162.3 ± 23.3	170.7 ± 21.6	168.5 ± 24.6
		Placebo	175.2 ± 20.9	172.3 ± 19.0	171.3 ± 18.5	176.4 ± 19.2	173.3 ± 19.2
Total protein (g/dL)	6.7-8.3	Test	7.6 ± 0.4	7.4 ± 0.2	7.4 ± 0.3	7.5 ± 0.3	7.4 ± 0.2
		Placebo	7.4 ± 0.3	7.3 ± 0.4	7.4 ± 0.2	7.4 ± 0.4	7.2 ± 0.4
Albumin (g/dL)	3.8-5.2	Test	4.8 ± 0.3	4.5 ± 0.4	4.5 ± 0.3	4.7 ± 0.3	4.6 ± 0.3
		Placebo	4.8 ± 0.3	4.7 ± 0.3	4.6 ± 0.3	4.7 ± 0.3	4.6 ± 0.3
Albumin/Globulin ratio	1.1-2.1	Test	1.8 ± 0.3	1.6 ± 0.3	1.6 ± 0.3	1.7 ± 0.3	1.7 ± 0.4
		Placebo	1.9 ± 0.3	1.8 ± 0.3	1.7 ± 0.3	1.8 ± 0.3	1.8 ± 0.3
Triglycerides (mg/dL)	50-149	Test	89.3 ± 28.2	92 ± 48.7	93.4 ± 36.4	92.7 ± 34.2	108.9 ± 50.5
		Placebo	80.6 ± 41.8	81 ± 55.8	95.2 ± 52.9	90 ± 49.7	102.8 ± 73.5
Total cholesterol (mg/dL)	150-219	Test	213.5 ± 34.0	208.1 ± 44.6	210.5 ± 36.5	211.9 ± 35.0	204.3 ± 36.4
		Placebo	206.1 ± 29.7	213.8 ± 37.9	211.9 ± 35.8	207.9 ± 36.5	205.3 ± 46.0
HDL-Cholesterol	F: 40-96	Test	66.1 ± 14.9	58.9 ± 15.5	62.7 ± 14.1	62.5 ± 13.1	61.3 ± 14.4
(mg/dL)	M: 40-86	Placebo	69.9 ± 12.4	69.8 ± 12.7	68.1 ± 12.5	67.6 ± 11.2	65.6 ± 9.6
LDL-Cholesterol (mg/dL)	70-139	Test	132.8 ± 31.7	129.6 ± 34.4	127.3 ± 27.9	132.2 ± 27.1	125.1 ± 29.0
		Placebo	121.9 ± 29.8	124.4 ± 37.4	121.6 ± 31.9	123.6 ± 34.4	121.6 ± 41.2
Free fatty acids (µEq/L)	150-600	Test	462.8 ± 254.7	449.2 ± 170.2	369.9 ± 144.7	482.1 ± 195.9	427.7 ± 176.5
		Placebo	477.4 ± 204.2	423.5 ± 190.1	380.9 ± 149.0	495.6 ± 212.8	454.3 ± 143.6
BUN (mg/dL)	8.0~20.0	Test	12.6 ± 2.5	11.2 ± 3.1	12.5 ± 3.0	12.5 ± 2.2	11.7 ± 2.8
		Placebo	11.9 ± 2.4	13.1 ± 3.2	10.8 ± 2.6	12 ± 2.6	11 ± 1.7
Uric acid	F: 2.5-7.0	Test	5.2 ± 1.2	5,1 ± 1.2	5.1 ± 1.0	5.2 ± 1.1	5.2 ± 1.1
(mg/dL)	M: 3.7-7.0	Placebo	5.6 ± 1.4	5.5 ± 1.3	5.2 ± 1.1	5.6 ± 1.3	5.5 ± 1.2
Creatinine	F: 0.47-0.79	Test	0.73 ± 0.2	0.74 ± 0.2	0.71 ± 0.2	0.76 ± 0.2	0.75 ± 0.2
(mg/dL)	M: 0.61-1.04	Placebo	0.8 ± 0.2	0.82 ± 0.2	0.82 ± 0.2	0.85 ± 0.2	0.84 ± 0.2
Na+ (mEq/L)	136-147	Test	141.7 ± 1.5	141.4 ± 1.5	141.8 ± 1.6	141.6 ± 1.7	142.4 ± 1.5
		Placebo	141.6 ± 1.2	142.2 ± 1.2	142.6 ± 1.2	141.5 ± 1.4	142.8 ± 1.9
K+ (mEq/L)	3.6-5.0	Test	4.9 ± 0.4	4.5 ± 0.4	4.5 ± 0.4	4.5 ± 0.4	4.2 ± 0.3 #
		Placebo	4.7 ± 0.5	4.6 ± 0.4	4.4 ± 0.2	4.1 ± 0.2	4.1 ± 0.2 #
Ca2+ mg/dL)	98-109	Test	9.2 ± 0.2	9.3 ± 0.3	9.1 ± 0.2	9.3 ± 0.2	9.3 ± 0.3
		Placebo	9.3 ± 0.3	9.4 ± 0.2	9.1 ± 0.2	9.3 ± 0.2	9.3 ± 0.2
Cl- (mEq/L)	95-107	Test	101.3 ± 1.7	100.8 ± 1.98	102.4 ± 1.0	101.1 ± 1.5	102.4 ± 1.7
		Placebo	100.3 ± 2.1	102 ± 1.8	103.3 ± 1.6	101.4 ± 1.7	103.4 ± 2.4 #
Inorganic phosphorus (mg/dL)	2.4-4.3	Test	3.3 ± 0.5	3.2 ± 0.5	3.3 ± 0.6	3.4 ± 0.5	3.2 ± 0.5
		Placebo	3.1 ± 0.5	3.1 ± 0.5	3.1 ± 0.4	3.0 ± 0.6	3 ± 0.6
Mg2+	1.8-2.6	Test	2.4 ± 0.1	2.3 ± 0.1	2.3 ± 0.1	2.3 ± 0.1	2.3 ± 0.1

(mg/dL)		Placebo	2.3 ± 0.1	2.3 ± 0.1	2.3 ± 0.2	2.3 ± 0.1	2.3 ± 0.2
Fe2+ (μg/dL)	48-154	Test	127.3 ± 51.3	105.6 ± 41.0	93.7 ± 23.4	95.9 ± 32.2	110.2 ± 46.1
		Placebo	118.1 ± 28.5	112 ± 47.6	100.6 ± 32.6	103.1 ± 30.3	115.9 ± 53.7
Blood glucose (mg/dL)	70-109	Test	95 ± 10.1	92.5 ± 6.8	93.9 ± 7.8	94.7 ± 9.1	92.7 ± 9.8
		Placebo	92.9 ± 5.4	96.6 ± 6.5	94.4 ± 5.9	96.9 ± 7.7	94.6 ± 7.5
HbA1c (%)	4.6-6.2	Test	5.3 ± 0.5	5.2 ± 0.5	5.2 ± 0.5	5.3 ± 0.5	5.5 ± 0.5
		Placebo	5.4 ± 0.3	5.2 ± 0.3	5.3 ± 0.3	5.3 ± 0.3	5.6 ± 0.2
Alkaline phosphatase (U/L)	38-113	Test	64.3 ± 21.9	65.4 ± 23.7	63.8 ± 21.6	64 ± 23.2	61.9 ± 21.0
		Placebo	69 ± 12.7	69.1 ± 12.9	69.4 ± 15.2	67.5 ± 14.0	65.3 ± 10.6
Total ketone bodies (μmol/L)	-130	Test	108.5 ± 141	126.8 ± 193.8	51.7 ± 17.3	122 ± 185.6	63.9 ± 15.6
		Placebo	88.9 ± 81	86.8 ± 114.4	42.6 ± 18.8	69.4 ± 46.2	42.1 ± 9.7
Acetoacetic acid (μmol/L)	-55	Test	21.9 ± 25.5	23.3 ± 29.5	12 ± 4.4	25.1 ± 27.4	13.3 ± 7.5
		Placebo	18.3 ± 15.8	17.9 ± 21.0	9.7 ± 4.0	17.4 ± 9.0	10.6 ± 3.2
3-Hydroxybutyric acid (μmol/L)	-85	Test	86.7 ± 116	103.5 ± 164.5	39.7 ± 14.0	96.9 ± 158.7	50.6 ± 38.4
		Placebo	70.6 ± 65.3	68.8 ± 93.5	32.9 ± 15.1	39.9 ± 31.2	31.4 ± 7.1
Lipoprotein (mg/dL)	-30	Test	24.4 ± 16.7	27.9 ± 16.0	26.8 ± 16.1	27.5 ± 16.4	24.5 ± 16.0
		Placebo	11.9 ± 7.5	13.1 ± 6.8	14.9 ± 8.1	15.6 ± 9.5	13.4 ± 8.1 *

Values are presented as mean ± SD. Dunnett's test was used for intragroup comparison with 0 week # $p < 0.05$. Unpaired t-test was used for intergroup comparison at each time point * $p < 0.05$.

Table S3. Hematological parameters from blood samples of participants

	Normal Range	Group	0w	4w	8w	12w	16w
			Mean ± SD	Mean ± SD	Mean ± SD	Mean ± SD	Mean ± SD
White blood cells	F: 3500-9100	Placebo	5780 ± 1692	6129 ± 1677	5693 ± 1731	5667 ± 1502	5713 ± 1541
(/μL)	M: 3900-9800	Test	5279 ± 1060	5631 ± 1112	5393 ± 1507	5764 ± 1548	5657 ± 1573
Red blood cells	F: 376-500	Placebo	477.5 ± 46.5	488.3 ± 58.9	477.9 ± 46.7	480.9 ± 57.8	470.5 ± 51.9
(×10^4/μL)	M: 427-570	Test	470.6 ± 31.2	485.7 ± 36.1	474.7 ± 31.7	475 ± 30.3	467.4 ± 28.4
Hemoglobin	F: 11.3-15.2	Placebo	15.1 ± 1.1	14.8 ± 1.5	15 ± 1.0	14.6 ± 1.3	14.7 ± 1.2
(g/dL)	M: 13.5-17.6	Test	15.2 ± 1.2	15.1 ± 1.3	15.3 ± 1.0	15 ± 1.1	14.9 ± 1.1
Hematocrit	F: 33.4-44.9	Placebo	44.4 ± 3.4	45.7 ± 4.3	44 ± 3.3	44.8 ± 4.4	44 ± 3.8
(%)	M: 39.8-51.8	Test	44.8 ± 3.0	46.4 ± 3.6	44.5 ± 3.0	46 ± 3.1	44.4 ± 2.8
MCV	F: 79.0-100.0	Placebo	93.1 ± 2.8	93.9 ± 3.5	92.2 ± 3.5	93.8 ± 2.6	93.7 ± 2.7
(fL)	M: 82.7-101.6	Test	95.2 ± 2.4	95.5 ± 2.4	93.8 ± 2.3	95.7 ± 2.2	95 ± 2.2
MCH	F: 26.3-34.3	Placebo	31.6 ± 1.6	30.5 ± 1.7	31.4 ± 1.5	30.6 ± 1.7	31.3 ± 1.6
(pg)	M: 28.0-34.6	Test	32.3 ± 1.4	31.2 ± 0.9	32.2 ± 1.0	31.2 ± 1.0	31.8 ± 0.9
MCHC	F: 30.7-36.6	Placebo	34 ± 1.0	32.5 ± 0.7	34.1 ± 0.9	32.6 ± 1.1	33.4 ± 0.8
(%)	M: 31.6-36.6	Test	33.9 ± 1.3	32.7 ± 0.6	34.4 ± 0.9	32.6 ± 0.9	33.5 ± 0.7
Platelet count	F: 13.0-36.9	Placebo	24.5 ± 5.0	26.8 ± 6.0	26.3 ± 6.7	25.6 ± 5.2	25.4 ± 5.6
(×10^4/μL)	M: 13.1-36.2	Test	23.5 ± 5.4	22.8 ± 3.4	23.7 ± 5.0	23.3 ± 4.0	22.8 ± 4.1

Values are presented as mean ± SD. Dunnett's test was used for intragroup comparison with 0 week # $p < 0.05$. Unpaired t-test was used for intergroup comparison at each time point * $p < 0.05$.

Table S4. Quantitative urine parameters of the participants

	Normal Range	Group	0w	4w	8w	12w	16w
			Mean ± SD	Mean ± SD	Mean ± SD	Mean ± SD	Mean ± SD
pH	5.0-7.5	Placebo	5.9 ± 0.5	6.1 ± 0.3	6.3 ± 0.5	6.3 ± 0.6	6.2 ± 0.5
		Test	6.0 ± 0.6	6 ± 0.6	6.3 ± 0.6	6.7 ± 0.8	6.4 ± 0.7
Specific gravity	1.005-1.030	Placebo	1.024 ± 0.007	1.02 ± 0.005	1.017 ± 0.005 #	1.018 ± 0.007#	1.019 ± 0.005
		Test	1.023 ± 0.008	1.021 ± 0.007	1.020 ± 0.008	1.021 ± 0.008	1.019 ± 0.007

Values are presented as mean ± SD. Dunnett's test was used for intragroup comparison with 0 week # $p < 0.05$. Welch`s t-test was used for intergroup comparison at each time point * $p < 0.05$.

Table S5. Qualitative urine parameters of the participants

	Normal Range	Group	0w						4w						8w						12w						16w					
			-	±	1+	2+	3+	4+	-	±	1+	2+	3+	4+	-	±	1+	2+	3+	4+	-	±	1+	2+	3+	4+	-	±	1+	2+	3+	4+
Protein	(-)	Placebo	14	1	0	0	0	0	12	0	1	0	0	0	15	0	0	0	0	0	15	0	0	0	0	0	15	0	0	0	0	0
		Test	14	0	0	0	0	0	14	0	0	0	0	0	14	0	0	0	0	0	12	2	0	0	0	0	12	2	0	0	0	0
Sugar	(-)	Placebo	15	0	0	0	0	0	13	0	0	0	0	0	15	0	0	0	0	0	14	0	1	0	0	0	15	0	0	0	0	0
		Test	14	0	0	0	0	0	13	0	0	0	0	0	14	0	0	0	0	0	13	0	0	0	0	0	14	0	0	0	0	0
Urobilinogen	(±)	Placebo	-	15	0	0	0	0	-	12	1	0	0	0	-	12	3	0	0	0	-	14	1	0	0	0	-	14	1	0	0	0
		Test	-	14	0	0	0	0	-	13	0	0	0	0	-	14	0	0	0	0	-	13	0	0	0	0	-	13	0	0	0	0
Occult blood	(-)	Placebo	15	0	0	0	0	0	13	0	0	0	1	0	14	1	0	0	0	0	15	0	0	1	0	0	13	1	0	1	0	0
		Test	14	0	0	0	0	0	13	0	0	0	0	0	14	0	0	0	0	0	13	2	1	0	0	0	13	6	0	0	1	0
Bilirubin	(-)	Placebo	15	0	0	0	0	0	13	0	0	0	0	0	15	0	0	0	0	0	15	0	0	0	0	0	15	0	0	0	0	0
		Test	14	0	0	0	0	0	13	0	0	0	0	0	14	0	0	0	0	0	12	1	0	0	0	0	14	0	0	0	0	0
Ketone bodies	(-)	Placebo	15	0	0	0	0	0	13	0	0	0	0	0	15	0	0	0	0	0	14	0	1	0	0	0	15	0	0	0	0	0
		Test	14	0	0	0	0	0	13	0	0	0	0	0	14	0	0	0	0	0	13	0	0	0	0	0	14	0	0	0	0	0

Values are presented as mean ± SD. Dunnett's test was used for intragroup comparison with 0 week # $p < 0.05$. Wilcoxon signed-rank sum test (Bonferroni correction) was used for intergroup comparison at each time point * $p < 0.05$.
